# The Role of Cerebral Embolic Protection Devices During Transcatheter Aortic Valve Replacement

**DOI:** 10.3389/fcvm.2018.00150

**Published:** 2018-10-23

**Authors:** Ozan M. Demir, Gianmarco Iannopollo, Antonio Mangieri, Marco B. Ancona, Damiano Regazzoli, Satoru Mitomo, Antonio Colombo, Giora Weisz, Azeem Latib

**Affiliations:** ^1^Interventional Cardiology Unit, Cardio-Thoracic-Vascular Department, San Raffaele Scientific Institute, Milan, Italy; ^2^Department of Cardiology, Hammersmith Hospital, Imperial College Healthcare NHS Trust, London, United Kingdom; ^3^Department of Cardiology, Montefiore Medical Center, New York, NY, United States; ^4^Division of Cardiology, Department of Medicine, University of Cape Town, Cape Town, South Africa

**Keywords:** stroke, embolic protection devices, aortic stenosis, TAVI, TAVR

## Abstract

Transcatheter aortic valve replacement is the therapy of choice for patients with severe aortic stenosis who have prohibitive or high surgical risk. However, the benefit of TAVR is attenuated by the occurrence of major disabling stroke which is associated with increased mortality and early-reduced quality of life. Despite advances in TAVR technology, stroke remains a serious complication that is associated with significant negative outcomes. The majority of these occur in the acute phase following TAVR where cerebral embolic events are frequent. Cerebral embolic protection devices (CEPD) have been developed to minimize the risk of peri-procedural ischemic stroke during TAVR. CEPD have the potential to reduce intraprocedural burden of new silent ischemic injury. In this review we outline the etiology and incidence of stroke in TAVR population, and systematically review current evidence for cerebral embolic protection devices.

## Introduction

Aortic stenosis (AS) is the most common valvular pathology in the elderly and its prevalence is expected to increase rapidly over the next decade due to an aging population (1, 2). Transcatheter aortic valve replacement (TAVR) has revolutionized the treatment of symptomatic AS with over 300,000 TAVR procedures performed worldwide, to-date. This resulted following a number of registries (3, 4) and randomized controlled trials (5–11) demonstrating mortality benefits of TAVR in inoperable and high-risk surgical patients. However, the benefit of TAVR is attenuated by the occurrence of major disabling stroke which is associated with increased mortality and in the short term reduced quality of life. Despite the evolution in TAVR technology, cerebrovascular events remain one of the most serious complications with long-term negative sequelae. Cerebral embolic protection devices (CEPD) have been developed to minimize the risk of peri-procedural ischemic stroke during TAVR. Furthermore, with the anticipated expansion of TAVR into intermediate risk and younger patients, the prevention of TAVR-related stroke and understanding the role of CEPD in this will become essential (12).

In this review we outline the etiology and incidence of stroke in TAVR population, and systematically review current evidence for cerebral embolic protection devices.

## Etiology

The temporal pattern of stroke following aortic valve intervention is similar between surgical and transcatheter aortic valve replacement. However, the main disparity between these occurs in the acute peri-procedural period with up to half of strokes occurring within the first 24-h after TAVR (13, 14). The PARTNER trial (6) observed a significantly increased risk of peri-procedural stroke (6.7%) compared to medical therapy (1.7%). Subsequent meta-analyses (15, 16) demonstrated 30-days stroke incidence of 3.1–3.3%, and that it confers a 3.5-fold increase in mortality at 1-year. After the initial 2 months, known as the late phase, there is a similar incidence of stroke between surgical and transcatheter aortic valve replacement groups that is likely to reflect the baseline risk profiles of the populations (13, 17).

### Acute stroke

Acute strokes are ischemic in the vast majority (95%) of patients and thought to be secondary to procedural factors (Table 1). These peri-procedural strokes occur due to embolization phenomenon arising from disruption of the vasculature, especially the aortic arch, degenerate aortic leaflets, or the left ventricular outflow tract. This causes calcific material or atheromatous plaque embolization (18). The passage of stiff guidewires, large caliber TAVR delivery systems, and prolonged procedural time have previously been associated with cerebral embolization in AS patients (19). Furthermore, repeated attempts to the cross calcified aortic valve, manipulation of the calcified aortic valve annulus, mechanical force of valve deployment, and pre- or post-dilatation may all be associated with further anatomical disruption leading to cerebral embolization. In addition, thrombotic cerebral microembolization has been observed in patients acutely following TAVR, potentially developing on guidewires or catheters. Lastly, CEPD studies have demonstrated the presence of myocardial tissue and plastic from TAVR delivery system as sources of cerebral embolization (18).

**Table 1:** Mechanisms of stroke in TAVR patients.

**Stroke timing**	**Mechanism of stroke**	**Possible associated factors**
Acute (Periprocedural)	Embolization phenomenon	- Wire or catheter manipulation in the aortic arch, ascending aorta or aortic arch - Crossing calcified aortic valve - Balloon aortic valvuloplasy - TAVR device manipulation across aortic root and annulus - TAVR prosthesis deployment - Postdilatation of TAVR
	Global ischemia	- Hemodynamic instability - Rapid ventricular pacing - Anesthetic complication
	Hemorrhagic	- Vascular complication - Anticoagulation (heparin) associated intraprocedurally
Subacute/Late	Thromboembolic	- Atrial fibrillation (new on-set or chronic) - Thromboembolic phenomenon (cardio-embolic)
	Hemorrhagic	- Long-term use of anti-coagulation and/or antiplatelet therapy

Hemodynamic instability occurring during TAVR can lead to systemic hypotension and consequently cerebral hypoperfusion. The effect of cerebral microemboli under these conditions is amplified, due to impairment of clearance and cementation of microemboli within small vessels (20). Rapid ventricular pacing constitutes the greatest risk of hemodynamic instability during TAVR, this is required during balloon valvuloplasty before or after TAVR, and in balloon expandable TAVR prostheses, or in cases where difficultly is encountered in precise positioning of self-expanding TAVR prostheses to minimize the risk of migration/embolization. In general, rapid ventricular pacing is well tolerated however in certain patients this can be associated with greater risk of hemodynamic instability, for example those with impaired left ventricular function or in those with marked left ventricular hypertrophy (17). Hemodynamic instability can also occur as a consequence of anesthetic complications, or secondary to hemorrhage.

## Subacute/late stroke

The etiology of delayed stroke after TAVR remains poorly characterized and is probably due to multiple factors, the primary cause thought to be thromboembolic. This can occur due to numerous reasons: valve crimping and balloon dilatation of prosthetic valve leaflets can cause structural damage that result in prothrombotic state with platelet and fibrin aggregation (14); the valve delivery system may scrape the diffuse atherosclerosis inside the aorta and some particles be dislodged later with the increased cardiac output and increased flow; increased risk of thrombus formation on the TAVR prosthesis as endothelialization can take over 1 year (21); and new onset atrial arrhythmias, predominantly atrial fibrillation, have been revealed to confer increased risk of ischemic stroke and mortality (22). In addition, TAVR prothesis leaflet thickening and leaflet thrombus have recently been reported following an acutely successful procedure. Makkar et al. (23) reported on reduced aortic-valve leaflet motion in 55 patients from a clinical trial of TAVR and two single-center registries that included 132 patients who were undergoing either TAVR or surgical aortic-valve bioprosthesis implantation. From the clinical trial arm, reduced leaflet motion was noted in 22 of 55 patients (40%) on computed tomography (CT) imaging approximately 1-month after TAVR. Of note, all the patients with reduced leaflet motion CT had hypoattenuating opacities noted in the corresponding leaflets on two-dimensional CT. The findings on transesophageal echocardiography were consistent with a hyperechogenic, homogeneous mass located on the aortic aspect of the prosthetic leaflets that prevented normal leaflet excursion. There was no significant difference between patients with reduced leaflet motion and those with normal leaflet motion with respect to the mean aortic-valve gradient. Importantly, there was no significant difference in the incidence of stroke or TIA between patients with reduced leaflet motion and those with normal leaflet motion in the clinical trial (2 of 22 patients and 0 of 33 patients, respectively; *P* = 0.16). However, in the pooled registries including surgical aortic valve replacement, a significant difference was detected (3 of 17 patients and 1 of 115 patients, respectively; *P* = 0.007). Chakravarty et al. (24) reported on 890 patients from two large registries who had CT imaging following either TAVR or surgical aortic valve replacement. It was demonstrated that 106 (12%) of 890 patients had subclinical leaflet thrombosis, including five (4%) of 138 with thrombosis of surgical valves vs. 101 (13%) of 752 with thrombosis of transcatheter valves (*p* = 0.001).Subclinical leaflet thrombosis was less frequent among patients receiving anticoagulants. Subclinical leaflet thrombosis resolved in 36 (100%) of 36 patients receiving anticoagulants, whereas it persisted in 20 (91%) of 22 patients not receiving anticoagulants (*p* < 0.0001). Although stroke rates were not different between those with (4·12 strokes per 100 person-years) or without (1.92 strokes per 100 person-years) reduced leaflet motion (*p* = 0.10), subclinical leaflet thrombosis was associated with increased rates of transient ischaemic attacks (TIAs; 4.18 TIAs per 100 person-years vs. 0.60 TIAs per 100 person-years; *p* = 0.0005) and all strokes or TIAs (7.85 vs. 2.36 per 100 person-years; *p* = 0.001). Although, CEPD have no role in the prevention of leaflet thrombosis, prosthetic valve thrombosis may potentially have a tangible deleterious effect on rates of cerebrovascular accidents, but further data from the randomized low risk TAVR trials are awaited which should help clarify this issue.

## Silent cerebrovascular events

The incidence of subclinical new cerebral ischemic lesions has been identified in as many as 93% of patients post-TAVR and recent pooled analysis reported an incidence of 77.5% (25). These are up to double of that seen in isolated surgical aortic valve replacement (26). Subclinical acute cerebral ischemic lesions can be accurately identified on diffusion-weighted magnetic resonance imaging (DW-MRI) with these regions demonstrating hyperintense signal as a result of reduction in water diffusion rate (27). Hyperintense signals on DW-MRI are well-established surrogate parameters for cerebral embolization and have already been investigated after catheter-based or cardiothoracic surgical interventions (28). In addition, cognitive function testing can be utilized to screen patients to determine those who may have had subclinical strokes (29). In various clinical contexts the occurrence of small brain infarcts has been linked to a higher incidence of stroke (30, 31) or cognitive impairment and dementia (32–34). Cerebral emboli detected on DWI-MRI increases the risk of clinically overt stroke by 2-4 fold and the greater the volume of lesions seen on DW-MRI, the greater the long-term risk of cognitive dysfunction and long-term dementia (32, 35). However, the prognostic significance of these subclinical brain injuries remains contentious and the correlation between new cerebral infarcts post TAVR and long-term cognitive decline or behavioral changes remain uncertain. Performing neurocognitive assessment immediately following TAVR is challenging in elderly patients since results can be influenced by the degree of alertness and fatigue, which is common in elderly patients peri-procedurally especially if sedation or general anesthetic have been administered (36). The BRAVO-3 MRI study investigated the role of intra-procedural parenteral anticoagulation (heparin vs. bivalirudin) during TAVR in reducing risk of cerebral emboli during TAVR (37). In this study, there was no difference in rates of with new cerebral emboli between the bivalirudin (54.5%) and heparin (58.1%) groups. Of note, all patients that presented with clinically overt stroke showed evidence of new emboli on MRI. The total volume of emboli, the volume of single embolus per patient, and the volume of the largest embolus per patient were higher in patients presenting with vs. without stroke at 30-days.

Transcarotid Doppler studies have shown a high incidence of high-intensity transient signal (HITS) throughout the entire TAVR procedure, especially during valve positioning and implantation (38), highlighting the high embolic risk during these phases of the procedure. Therefore, the use of CEPD might play an important role during these at high risk phases of the TAVR procedure. All studies evaluating CEPD have focused on the assessment and characterization of new brain ischemic lesions on DW-MRI as the main efficacy endpoints (39). The relatively small incidence of clinically apparent cerebrovascular events makes them difficult to use as endpoints in clinical trials, shifting the attention to subclinical cerebral injury (17).

## Cerebral embolic protection devices

**Figure 1:**
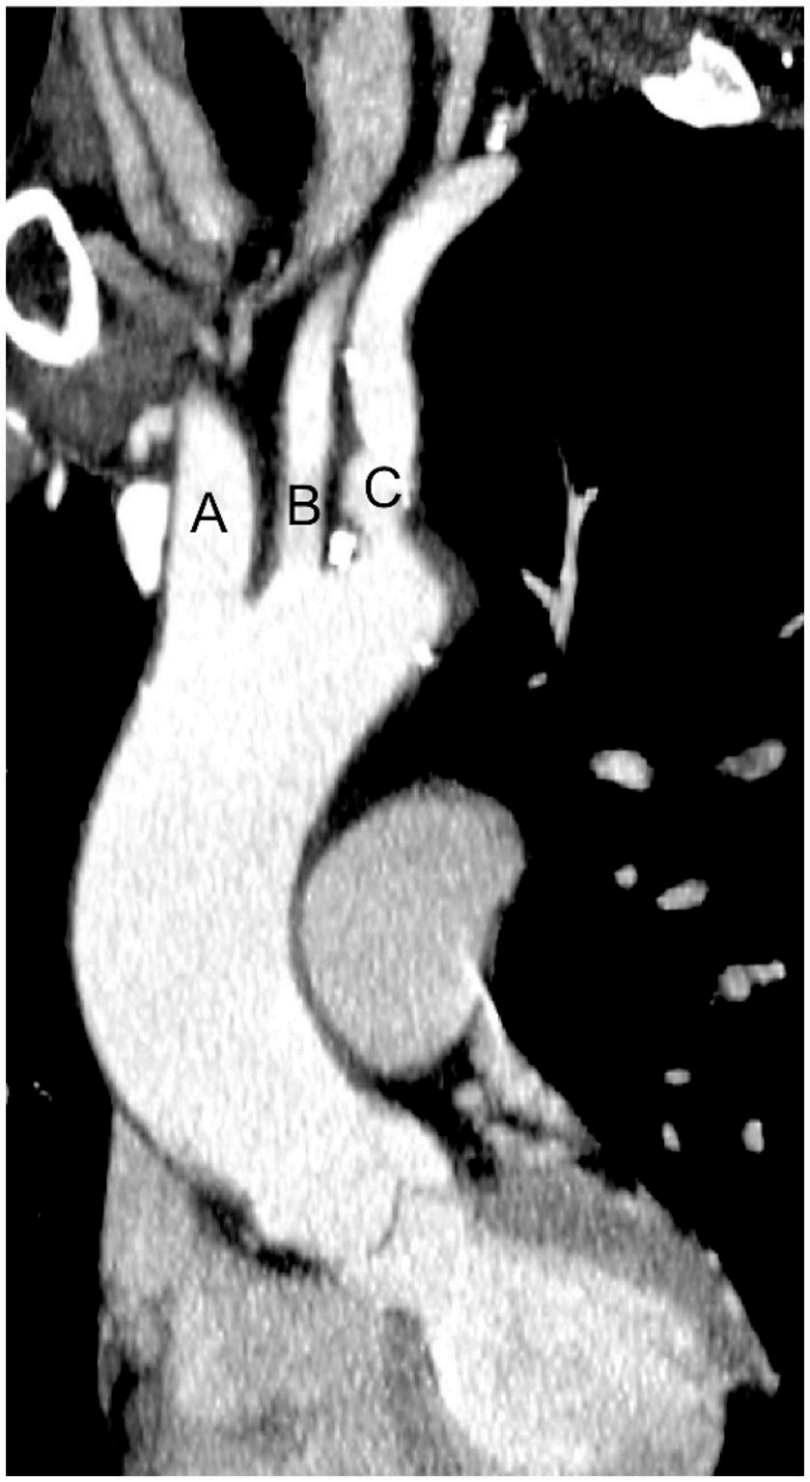
Computed tomography image of ascending aorta and supra-aortic vessels. **(A)** Brachiocephalic artery; **(B)** Left common carotid artery; **(C)** Left subclavian artery.

Cerebral embolic protection devices are filters designed to capture or deflect emboli traveling to the brain during TAVR procedures in order to protect the supra-aortic vessels from embolic debris. These filters are normally positioned across the origin of supra-aortic vessels before the advancement of the TAVR system across the aortic valve and is retrieved at the end of the procedure (Figure 1). The positioning of these devices can be challenging particularly if atherosclerotic plaques are located in the vicinity of the ostium of supra-aortic vessels or aortic arch, hampering the implantation and positioning of CEPD which may even promote plaque disruption and consequently cerebral embolization (40). Initial in-human experiences have shown the feasibility and safety of CEPD during TAVR (41, 42). Currently there are three devices commercially available with studies that have evaluated their efficacy (Table 2).

**Table 2:** Cerebral protection devices and current evidence base.

	**Embrella**	**Claret**	**TriGuard**
Manufacturer	Edwards Lifesciences; Irvine, California, United States	Claret Medical, Inc.; Santa Rosa, California, United States	Keystone Heart Ltd., Herzliya, Israel
Structure	Oval shaped nitinol frame (length 59 mm, width 25.5 mm) Covered with a porus polyurethane membrane Pore size: 100 μm	Two oval coned mesh positioned within brachiocephalic (sized 9–15 mm diameter) and left common arteries (sized 6.5–10 mm in diameter) Pore size: 140 μm	Single-wire nitinol frame and mesh filter, maintained by stabilizers in the brachiocephalic artery and the inner curvature of the aortic arch. Pore size: 130 μm
Delivery approach	Radial/brachial artery	Radial/brachial artery	Femoral
Sheath Size	6 French	6 French	9 French
Primary Mechanism	Deflection	Filter and capture	Deflection
Coverage	Brachiocephalic and the left common carotid arteries	Brachiocephalic and the left common carotid arteries	Brachiocephalic, left common carotid, and left subclavian arteries
Most relevant study	PROTAVI-C (41)	SENTINEL (43)	DEFLECT III (44)
Methods	Prospective, non-randomized study. Device *n* = 54 Control *n* = 12	RCT Safety arm *n* = 123 Device arm *n* = 121 Imaging control arm *n* = 119	RCT Device *n* = 46 Control *n* = 39
Patient and procedural characteristics	52% male, median age 83 years. Only balloon expandable TAVR (Edwards Sapien XT) Only Transfemoral TAVR Successful device positioning in 100%	48% male, medial 83 years Balloon expandable TAVR in 70% Transfemoral TAVR in 95% Successful device positioning in 94%	46% male, mean age 82 years Balloon expandable TAVR in 64% Transfemoral TAVR in 97% Successful device positioning in 89%
Outcomes	**DW-MRI:** - Non-significant increase in lesion numbers (8 vs. 4, *P* = 0.41) in device group. - Significantly lower lesion volumes (40% smaller, *P* = 0.003) in device group. **TCD:** - Higher procedural HITS rates in device group.	**DW-MRI:** Protected territories: - 42% reduction in device arm of total lesion volume (*P* = 0.33) - 33% reduction in number (*P* = 0.90). All territories: - 5% reduction of total lesion volume (*P* = 0.81), 40% in number (*P* = 0.77). Neurocognitive: - no difference in overall composite scores at baseline, 30 days, or 90 days. - Change in neurocognitive scores from baseline to 30-day follow-up correlated with median new lesion volume in protected territories	**DW-MRI:** - Device related greater freedom from new cerebral DWI lesions (21.2 vs. 11.5%), - 44% reduction of median lesion size **Neurocognitive**: - Reduction worsening in National Institutes of Health Stroke Scale score from baseline (2.6 vs. 12.1%) in device arm
Ongoing studies	No registered on-going study	Ongoing study powered for efficacy (PROTECT-TAVI Trial; ClinicalTrials.gov Identifier: NCT02895737)	Ongoing study powered for efficacy (REFLECT Trial; ClinicalTrials.gov Identifier: NCT02536196)

## Embrella device

The Embrella Embolic Deflector device (EED) (Edwards Lifesciences; Irvine, California, United States) is a filter designed to deflect debris traveling to the brain during the positioning and implantation of the TAVR valve. The distal end of the deflector consists of an oval shaped nitinol frame (length 59 mm, width 25.5 mm) covered with a porus polyurethane membrane (100 microns pore size). The frame of the device has two opposing petals that are positioned along the greater curve of the aorta, covering the ostia of both the brachiocephalic and the left common carotid arteries (45). The device is inserted via the right radial or brachial approach using a 6-French delivery system. The EED system is deployed at the beginning of the TAVR procedure just before any attempt to cross the native aortic valve. Nietlispach et al. reported the first in-human experience with the EED device showing the feasibility and safety of device implantation in a preliminary series of 4 patients (1 aortic valvuloplasty, 3 TAVR procedures) (41). Subsequently, the PROTAVI-C study (45) evaluated the procedural safety, technical feasibility, and exploratory efficacy of the EED. This prospective non-randomized study included 54 patients, with 42 patients receiving the EED device and 12 patients not receiving it (control group). TAVR procedures were performed by transfemoral approach with Edwards Sapiens XT. The PROTAVI-C study demonstrated that EED use during TAVR is feasible and safe with minimal procedural complications related to the device (1 radial thrombosis with no clinical consequences and 1 pseudoaneurysm of the brachial artery that required surgical repair). The EED system did not prevent the occurrence of cerebral microemboli during TAVR as evaluated by transcranial Doppler during the procedure. The number of HITS was actually higher in the EED group than in the control group, 632 [interquartile range, 347-893] vs. 279 [interquartile range, 0–505], respectively (*p* < 0.001). Therefore, suggesting that EED manipulation may also represent a potential source of embolic debris. In addition, the use of EED had no effect on the occurrence and number of new ischemic lesions as evaluated by DW-MRI at 7 days after the procedure. These ischemic lesions disappeared within few weeks (as evaluated by DW-MRI at around 30 days) and were not associated with any neurological and cognitive impairment. However, the use of a EED was associated with a reduction in lesion volume compared to the control group. Fundamentally, this study was limited by the low number of patients and lack of randomization. However, the EED device is currently not available commercially.

## Claret device

The Claret embolic protection device (CD) (Claret Medical, Inc.; Santa Rosa, California, United States) is designed to capture debris dislodged during TAVR and it is the first device with FDA approval (46). The system consists of a dual filter system deployed via the right radial or brachial approach to the brachiocephalic and left common carotid arteries. It consists of a proximal filter (sized 9–15 mm in diameter) delivered in the brachiocephalic artery covering all areas of the brain supplied by the right vertebral and right carotid artery and a distal filter (sized 6.5–10 mm in diameter) delivered in the left common carotid artery. The left vertebral artery, which usually originates from the left subclavian artery, remains unprotected, as does the cerebral regions fed by this vessel. At the start of the procedure the system is advanced through a 6F sheath and it is deployed in the aortic arch and withdrawn following removal of the TAVR delivery system (42). The CLEAN TAVI study (43) was a single center, blinded, randomized clinical trial that evaluated the efficacy of the Claret device in reducing the number of cerebral lesions in patients undergoing TAVR with Medtronic CoreValve. The primary endpoint was the reduction in number of lesions on DW-MRI at 2 days post-TAVR. The secondary outcome was the difference in volume of new lesions after TAVR in potentially protected territories. The study included 100 patients randomized 1:1 to the control or filter group. This showed a reduction in the number of new ischemic cerebral lesions [difference 5.00 (IQR, 2.00-8.00); *p* < 0.001] and volume of cerebral lesions in the filter group compared to the control group [difference 234 mm^3^ (95% CI, 91-406); *p* = 0.001]. These changes were observed largely within cerebral territories that were protected by the filter (day 2 post-TAVR: 246 vs. 527 mL, *p* = 0.002). The MISTRAL-C study (47), a multicenter, double-blind, randomized trial that confirmed the efficacy of CD in reducing the number of new ischemic cerebral lesions and the volume of these lesions in 65 patients randomized 1:1 to CD vs. non-CD. The main limitation of this study is that only 57% of the randomized patients underwent follow-up DW-MRI. The SENTINEL trial (48) is the largest randomized clinical trial evaluating the safety and efficacy of a transcatheter CD system during TAVR. The SENTINEL trial enrolled 363 patients, who were randomized 1:1:1 into a safety arm (*n* = 123), an imaging device arm (*n* = 121), and an imaging control arm (*n* = 119). In this study a significant reduction of median total new lesion volume in protected territories, evaluated by DW-MRI 2-7 days after TAVR, was not observed (102.8 mm^3^, IQR 36.9-423.2 mm^3^ in the device arm vs. 178.0 mm^3^, IQR 34.3-482.5 mm3 in the control arm; *p* = 0.33), However, the use of the Sentinel device during TAVR was safely performed and histopathological debris was found within filters in 99% of patients, confirming the embolic risk during TAVR with frequent embolization of non-thrombotic material (vascular material in 94% of cases). Importantly, it was demonstrated for the first time that there is a correlation between new lesion volume and neurocognitive decline. Latib et al. (36) identified some challenges related to this trial that can be extended to other CEPD trials. Firstly, the need of baseline MRI to detect previous neurological damage and their impact on new cerebral lesions. *Post-hoc* multivariable analysis in the SENTINEL trial identified pre-existing lesion volume as main predictor of new lesion volumes. In addition, after adjusting for baseline T2/FLAIR lesion volume, there was a reduction in new lesion volume in both protected and all territories in the device vs. control arms. Secondly, the time points of evaluation of 2–7 days after TAVR might create too much heterogeneity in terms of detected volumes of ischemic lesions because of the time-dependent sensitivity of DW-MRI. As a matter of fact, the time point for performing DW-MRI post-TAVR could affect the sensitivity for detecting silent cerebral infarcts, as these lesions tend to disappear over time, being totally absent at 30 days following the procedure (49, 50). Thirdly, the evaluation of cognitive dysfunction might be misleading in elderly patients in the first few days after TAVR, therefore a simpler and more focused battery of tests may be repeated later in time. Furthermore, the Claret device can only protect completely 9 out of 28 brain territories because of the dual blood supply of the posterior circulation. Thus, if we believe that cerebral protection is important, it is not acceptable that these areas of the brain remain unprotected. The even embolic distribution shown on DW-MRI validates the need of a comprehensive brain protection (51, 52).

## Triguard device

The TriGuard (TG) CEPD (Keystone Heart Ltd., Herzliya, Israel) is a mechanical system designed to deflect cerebral emboli during TAVR while allowing maximal blood flow to the brain and it is the only deflection device that covers all 3 cerebral vessels. The device is a single-wire nitinol frame and mesh filter with pore size of 130 μm and it is positioned across all 3 cerebral vessels and maintained by stabilizers in the innominate artery and the inner curvature of the aortic arch. At the start of the TAVR procedure, a 9 Fr arterial sheath is inserted in the contralateral femoral artery through which the TriGuard device is advanced to the aortic arch and deployed to cover the ostia of the three major cerebral vessel take-offs and it is withdrawn after completion of the TAVR procedure (44).

The DEFLECT I (44) and DEFLECT II (53) studies are single arm studies that confirmed the safety and performance of the first and second generation TriGuard device. In particular, the DEFLECT I formed the basis for TriGuard been granted CE mark in October 2013. The DEFLECT III (54) a prospective, multi-center, single-blind, randomized controlled trial evaluating the safety, efficacy and performance of the TriGuard device in subjects undergoing TAVR. The study included 85 patients randomized 1:1 to CEDP (46) or unprotected TAVR (39). This study showed that embolic protection during TAVR with TriGuard was safe and complete vessel coverage was achieved in 89% of the patients. The safety endpoint (including death, stroke, life-threatening or disabling bleeding, stage 2 or 3 acute kidney injury, or major vascular complications) was not different between the two groups. However, in the intention-to-treat analysis the use of TriGuard was associated with a greater freedom from new cerebral DWI lesions (21.2 vs. 11.5%), a reduction in “new neurologic impairment” defined as worsening in National Institutes of Health Stroke Scale score from baseline (2.6 vs. 12.1%) and a reduction in single and multiple lesions volume, especially for lesion volume of small and medium size (< 150 mm^3^). Additionally, it showed improved cognitive function in some domain at discharge: in the International Shopping List Test (a measure of episodic memory), significant differences were observed when patients were evaluated at discharge, favoring the interventional arm (65.4 vs. 30.4%, *p* = 0.022). The main limitations of this study are its lack of statistical power to evaluate the safety and efficacy endpoints and the high loss to follow-up (31% were lost to the post-interventional DWI evaluations and 26% were lost to the post-interventional cognitive and neurologic assessments). Lansky et al. (54) performed a pooled analysis on DEFLECT I, DEFLECT II studies, and the Neuro TAVR registry, this was a registry of 142 patients undergoing TAVR with TriGuard protection (*n* = 59) vs. no protection (*n* = 83). The study reported that TriGuard protection significantly reduced the incidence of in-hospital Valve Academic Research Consortium-2–defined stroke (VARC-2) (0 vs. 6%; *P* = 0.05), the incidence of stroke as defined by worsening National Institutes of Health Stroke Scale (NIHSS) with DW-MRI lesions (0 vs. 19%; *P* = 0.002), brain embolic lesion volume on MRI (315 + 620 mm3 vs. 511 + 893 mm3; *P* = 0.04) and demonstrated improved functioning on cognitive testing post-TAVR. The REFLECT-US (55) trial is an ongoing multicenter, randomized controlled trial evaluating the safety, efficacy, and performance of the TriGuard device in a larger cohort of patients undergoing TAVR compared to previous studies. This trial is including 285 TAVR subjects with 2:1 randomization to TriGuard (190 patients) and unprotected TAVR (95 patients).

## New cerebral protection devices

Several new CEPD are currently under development or early first-in-human analysis, these are outlined in Table 3. The Emblok Embolic Protection System (Innovative Cardiovascular Solutions, LLC) is one of the devices that has ongoing clinical feasibility study. This device provides full circumferential coverage of the aortic arch, hence protecting all supra-aortic vessels, utilizing a pore size of 125 μm. The system incorporates an integrated 4-Fr radiopaque pigtail catheter which provides constant visualization, from which aortagram can be performed both for CEPD and TAVR deployment. In addition, the radiopaque pigtail catheter aids in defining the non-coronary cusp hence it facilitates precise valve implantation while potentially decreasing contrast injections during TAVR positioning and deployment. The delivery system is 11–Fr compatible and allows two devices (i.e., embolic filter and pigtail catheter) to be deployed through a single access site supported by 0.035 guidewire. Clinical studies are currently underway to establish the safety and efficacy of Emblock in TAVR patient. In addition, there are other CEPD that currently under development or early first-in-human analysis, these are outlined in Table 3.

**Table 3:** Percutaneous cerebral protection devices currently under development or first-in-man study stage.

	**Emblock**	**Point-Guard**	**Emboliner**	**ProtEmbo**	**Embolisher**	**Fliterlex**
Device Illustration	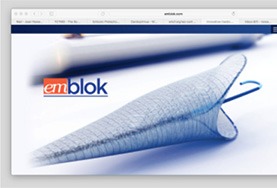	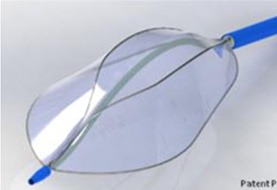	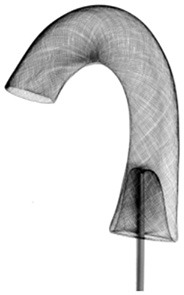	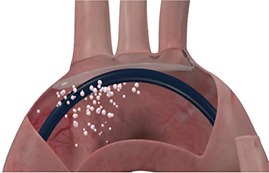	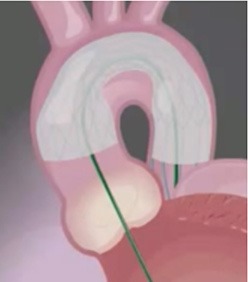	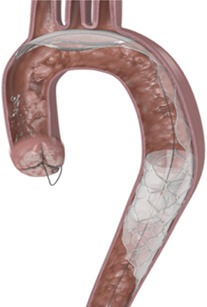
Company	Innovative Cardiovascular Solutions	Transverse Medical	Cardiological Solutions	Protembis	Cardioptimus	Fliterlex
Regulatory status	Feasibility study ongoing	Pre-clinical	Pre-clinical	Feasibility study awaited	Pre-clinical	Pre-clinical
Access	12F Contralateral Transfemoral	Unclear (Assume femoral)	9F Contralateral Transfemoral	6F left Transradial	Contralateral Transfemoral	Ipsilateral Transfemoral
Embolic protection mechanism	Capture and removal	Deflector, capture and removal	Capture and removal	Deflector	Deflector	Deflector
Cerebral Protection	All supra-aortic arteries	All supra-aortic arteries	All supra-aortic arteries	All supra-aortic arteries	All supra-aortic arteries	All supra-aortic arteries
Positioning	Aortic Arch	Aortic arch	Aortic arch	Aortic arch	Aortic arch	Aortic arch and descending aorta
Other features	• Integrated pigtail catheter • Designed to minimize use of contrast	• Sealing technology • Conforms to aortic arch	• Dual-layer Nitinol mesh filter mounted on a 6-Fr catheter	• Deflection of microparticles as tiny as 60 microns to descending aorta		• Full protection: Brain, Aorta and Body (kidney)

## Discussion

Despite advances in TAVR technology, stroke remains a serious complication that is associated with significant negative outcomes. The majority of these occur in the acute phase following TAVR where cerebral embolic events are frequent. CEPD have the potential to reduce intraprocedural burden of new silent ischemic injury. Although individual CEPD studies have not yet demonstrated a reduction in rates of silent cerebral ischemic lesions evaluated by DW-MRI, they have demonstrated reductions in total cerebral ischemic volumes. Giustino et al. (56) performed a meta-analysis on 5 studies evaluating the efficacy of these 3 CEPD. A total of 625 patients were included, 376 with CEPD and 249 without CEPD, the CEPD group showed a lower risk in death or stroke, suggesting that CEPD may be a clinically relevant adjunctive strategy in patients undergoing TAVR. Aufrett e al. meta-analysis (sixty-four studies involving 72,318 patients) reported that female sex, chronic kidney disease, enrollment date, and new-onset atrial fibrillation were predictors of early CVE after TAVR. The main limitation of this meta-analysis is that most of the studies included were not powered for CVEs as main endpoint (57). A very large multicentre study, powered for early CVEs, is needed to identify which are the predictors of such events that will help us tailoring our preventive strategies. Preventing procedure-related cerebral injury remains a significant unmet clinical need with potentially important long-term sequelae. As we move to low-risk patients, the bar to ensuring good TAVR outcomes will become much higher. Hence, CEPD could potentially become standard of care if: (a) we accept as a community that silent cerebral infarction has a negative impact on long-term outcomes and that prevention of these is as important as preventing stroke; (b) CEPD devices are easy to use, safe, provide full protection of supra-aortic arteries during the procedure, and can be rapidly implanted without adding significant time to the procedure or interfering with valve positioning or deployment; (c) specific reimbursement for CEPD during TAVR become available and/or the cost of devices decrease significantly. If the previous conditions are satisfied, a very large multicentre randomized study might be conducted to evaluate the clinical benefit from CEPD devices.

## Conclusion

Stroke remains one of the most serious complication following TAVR with associated worse outcomes that negate the benefit of TAVR procedure. Although CEPD have been demonstrated to reduce cerebral infarct volume, whether it decreases rates of both silent cerebral ischemic lesions and clinically evident ischemic strokes remains unclear. However, there will be greater emphasis on prevention of cerebral ischemic events as we move to low-risk patients. To elucidate the exact role of CEPD, a large randomized controlled trial with long-term follow-up with baseline and follow-up cerebral MRI imaging, and full neurological clinical evaluation, ideally using a device that protects all supra-aortic arteries, to establish the role of CEPD both in the short and long term.

## Author contributions

OD, GI, GW, and AL substantially contributed conception and design of the manuscript. OD and GI wrote the first draft of the manuscript. All authors contributed to manuscript revision, read and approved the submitted version.

### Conflict of interest statement

The authors declare that the research was conducted in the absence of any commercial or financial relationships that could be construed as a potential conflict of interest.
